# Supplementary material for: The effect of various metal-salts on the sedimentation of soil in a water-based suspension
Source: PLoS One. 2020 Jan 14;15(1):e0227338. doi: 10.1371/journal.pone.0227338 (PMC6959554; doi:10.1371/journal.pone.0227338)
Supplement: Attachment — Submitted filename: Responses.docx [file pone.0227338.s002.docx]

Author response to Editor and Reviewer comments - PONE-D-19-17683: *The effect of various metal-salts on the sedimentation of soil in a water-based suspension*

Dear Editor

Please find our responses to all comments made by Yourself and the reviewers. We appreciate the constructive, helpful comments, considered all of them, and thrived to implement them. However, in very few cases we think the suggestion or request was irrelevant to the manuscript, or we felt that we already covered the topic there or elsewhere in the manuscript, and hence provided a response accordingly for your consideration. We used italics for our responses.

Also, we would like to clarify that one of the authors name shoudl be corrected from Victori Labancz to Viktoria Labanz.

We are looking forward to hearing from you.

Kind regards,

Andras Sebok on behalf of all co-authors

Comments by the Editor:

1. In your Methods section, please including geographic coordinates for the data set if available.

*Coordinates to the origin of the soil material are now included in the manuscript.*

2. We note that you have included the phrase “data not shown” in your manuscript. Unfortunately, this does not meet our data sharing requirements. PLOS does not permit references to inaccessible data. We require that authors provide all relevant data within the paper, Supporting Information files, or in an acceptable, public repository. Please add a citation to support this phrase or upload the data that corresponds with these findings to a stable repository (such as Figshare or Dryad) and provide and URLs, DOIs, or accession numbers that may be used to access these data. Or, if the data are not a core part of the research being presented in your study, we ask that you remove the phrase that refers to these data.

*We made the presentation of pH data to be part of the manuscript now (Figure 4 and relevant discussion) and included other previously referred individual information in the text.*

3. Please ensure that you refer to Figure 6 in your text as, if accepted, production will need this reference to link the reader to the figure.
Additional Editor Comments (if provided):

*Figure 6 is now replaced. We are discussing results of statistical analysis, and the stat tables themselves are planned to be made supplementary information. (S1 Appendix) (All such references point at the CHANGE-TRACKED version of the uploaded revised manuscript.*

Authors need to work on further in this manuscript to make it in a desired shape.
Both reviewers are partially satisfied and suggested number of changes for the clarity and betterment of the manuscript. Hence authors are suggested to address the issues raised by the reviewers for further consideration in journal.

*We have attempted to address all comments and generally improve cohesion in the manuscript’s presentation. We hope it helped improve the manuscript to a desirable degree.*

Reviewer comments to the Authors

1. Is the manuscript technically sound, and do the data support the conclusions?

The manuscript must describe a technically sound piece of scientific research with data that supports the conclusions. Experiments must have been conducted rigorously, with appropriate controls, replication, and sample sizes. The conclusions must be drawn appropriately based on the data presented.

Reviewer #1: Partly
Reviewer #2: Partly

*We addressed the reviewers’ and the Editor’s concerns in detail – we hope these changes will respond to the relevant concerns.*

2. Has the statistical analysis been performed appropriately and rigorously? 

Reviewer #1: No
Reviewer #2: No

*We now present a statistical analysis of the results. We hope these changes will respond to the relevant concerns. Please see newly inserted text (Lines 254-272,291-299 and S1 Appendix), and the analysis itself uploaded/intended as supplementary information.*

3. Have the authors made all data underlying the findings in their manuscript fully available?

The [PLOS Data policy](http://www.plosone.org/static/policies.action#sharing) requires authors to make all data underlying the findings described in their manuscript fully available without restriction, with rare exception (please refer to the Data Availability Statement in the manuscript PDF file). The data should be provided as part of the manuscript or its supporting information, or deposited to a public repository. For example, in addition to summary statistics, the data points behind means, medians and variance measures should be available. If there are restrictions on publicly sharing data—e.g. participant privacy or use of data from a third party—those must be specified.

Reviewer #1: Yes
Reviewer #2: Yes

*We have removed references to non-published data, and included a figure that resents multiple types of information – among them pH and its change during the experiments. Support to the statistical statements is being made available as supplementary information.*

4. Is the manuscript presented in an intelligible fashion and written in standard English?

PLOS ONE does not copyedit accepted manuscripts, so the language in submitted articles must be clear, correct, and unambiguous. Any typographical or grammatical errors should be corrected at revision, so please note any specific errors here.

Reviewer #1: Yes
Reviewer #2: Yes

*Thank you for the positive comment. The cited typos and noted complex sentences have been corrected/altered and the manuscript re-read and edited.*

5. Review Comments to the Author

Please use the space provided to explain your answers to the questions above. You may also include additional comments for the author, including concerns about dual publication, research ethics, or publication ethics. (Please upload your review as an attachment if it exceeds 20,000 characters)

Reviewer #1: Review report on “The effect of various metal-salts on the sedimentation of soil in a water-based suspension” (PONE-D-19-17683)
The present article evaluated the efficiency of different combinations of cations and anions at different concentrations for speedy sedimentation of suspended soil particles under static condition with a goal to upscale it and employ it in sedimentation ponds to reduce soil and associated nutrient loss to water bodies after erosion. In general, the manuscript is well written. Authors have also very aptly pointed out the limitations and boundary conditions of the present study. However, the manuscript has to be improved keeping in mind the following comments before it can be accepted for publication:

*We appreciate the constructive and generally positive comments. We hope we managed to address all the concerns raised by Reviewer #1.*

Major comments
1) Line 57-59: “Nutrients tend to bind stronger to these smaller particles due to their greater relative surface area and by that their greater adsorption capacity ([8], [9]).”- I think the greater adsorption capacity of small sized (< 2 µm) soil particles is mainly due to their charge characteristics, rather than the size itself. In the entire manuscript, the main focus has been on size of particles, but sedimentation of colloidal particles due to addition of excess cations or anions is due to suppression of diffuse double layer, a charge characteristic. This point should also be mentioned properly in ‘Introduction’ and ‘Discussion’ sections.

*Our work was motivated by the understanding that masses of soil particles and bound/absorbed nutrients erode into surface water bodies – causing physical and chemical pollution to the environment, and an economic loss.*

*In the literature review we pointed at the fact – without providing details of the otherwise well described mechanism – that smaller particles tend to bind more nutrients. This is essentially the reason why we repeatedly refer to the size of particles: what is important functionally is that smaller particles bind more nutrients while those are the most difficult to sediment.*

*We do not dispute the reviewer’s point that the mechanism to the improved sedimentation effect by salts is through the suppression of the diffuse double layer, but we think that this is not more relevant to the overall message of the paper than the degree to which we already introduced the phenomenon as the driving mechanism. (Lines 59-61, 91-94) We believe that if the subject of our research was related to nutrient cycling, the phenomenon would be more relevant to discuss in detail, its specific role towards sedimentation does not require more details.*

2) Line 114: “10, 20, 40 and 80 mg/L”- It is not clear whether these concentrations are with respect to the soil suspension or the solution of salts added? If these are salt solution concentrations then how much mL of salt solution was added to 500 mL suspension?

*We have referred to this in the text, but now we made it even more explicit in e.g. L 133 and checked elsewhere. We were referring to the concentration in the final solution.*

3) Table 1: NaOH and Ca(OH)2 are not salts, they are strong alkalis. These two are supposed to increase the pH of suspension, and consequently the pH dependent negative charges on colloidal particles. What was the logic behind using these two? How much was the pH increase due to addition of these two?

*We had referred to them in their dry form – which is now revised. The logic in using them in our work was to provide a more complete picture of the anion-cation matrix that are otherwise available. Ca(OH)2 even has practical environmental application such as in soil improvement and water treatment.*

*As suggested, we also expected – and logged – the pH response, which is now shown and discussed in Figure 4 and the related discussion.*

4) Line 169-173: Please explicitly mention the equation as well show how ‘t’ times ‘ln2’ gives ‘half-time’ to make it more intelligible.

*We have attempted to make this more intelligible – thank you.*

5) Table 3: Authors could consider to run a two-way ANOVA taking ‘type of salt’ and ‘concentration of salt’ as two factors to show their individual effect on sedimentation as well as to identify the most appropriate concentration of the most suitable salt for achieving required sedimentation efficiency. In that case, there is no need to provide replication wise data; mean values with standard error of mean along with group letters based on Fisher’s LSD or Tukey’s HSD would be fine. Accordingly one sentence from this table should be added to ‘Conclusions’.

*We understood and appreciate the comment and need for more objective support. We note, however, that we found that the suggested two-way ANOVA was not very useful. Instead, we separated cations and anions, and along with concentration as the third factor, we ran three-way ANOVA. We then used the embedded F test and interpretation, as well as followed up with t-tests of select salt-pairs that (1) had potential future applicability, and/or (2) the difference could not automatically be explained by known rules, such as the Shulze-Hardy rule. Presentation of the interpretations are on Lines 254-272,291-299, and the support tables are made available as supplementary information (S1 Appendix).*

6) Line 287-292: What about the dispersion that is likely to be promoted by addition of a strong alkali like NaOH?

*We noted, thank you, and included this factor in the discussion. Figure 4 presents a case where this effect is likely seen: addition of more concentrated NaOH increases the removal efficiency, but that efficiency is still under the control’s which we understand is likely due to the dispersion effect. Else the ionic charge effect would make the efficiency to be over that of the control’s.*

Minor comments
1) Line 46-48: “The ponds’ efficiency varies greatly, however, and depends on many factors, among them the sediment type, the pond’s dimensions and its nominal hydraulic loading ([4], [5]).”- Re-write this sentence to make it more meaningful. I think authors wanted to point out that factors like “sediment type, the pond’s dimensions and its nominal hydraulic loading” are more important than others.

*This is now rewritten.*

2) Line 100: It should be 13.9% instead of 13,9%
3) Please use ‘L’ instead of ‘l’ to indicate ‘Litre’ throughout the manuscript
4) Line 186: It should be ‘comprehensible’ instead of ‘comprehendible’
5) Table 2: Please correct the “ before half-time
6) Line 331: Is it 250.000 or 250,000? Please check
7) Line 351-352: Please correct the values. There are commas instead of decimal points.

*These are now rewritten.*

Reviewer #2: Although the MS is reviewed for a very small number of days. I have certain queries that has to be resolved before it is finally accepted in PLOS One.

*Thank you for the positive consideration and comments. We tried to address them all.*

1. Line 45-46- Reports ...2 and 3. Reframe the sentence for clarity.
2. Line 47-48- The ponds efficiency...4,5. Kindly reframe it as “among them are”.
3. Line 57- It is Brownian motion. So change it accordingly across the MS.

*These are now rewritten.*

4. Line 74-76- Under reductive condition...disappear over time. The statement is not clear. Kindly elaborate what are the bonds you want to explain here. Secondly what is the redox potential at this point.

*The statement refers to secondary binding forces in soil by e.g. organic compounds and iron and manganese, which will weaken with more reductive conditions. We found it important to refer to the reductive conditions due to the potential fragmentation and production of smaller particles. We measured redox potential during the experiment, but during the short experiments did not find useful trends in them, which we simply refer to in a statement.*

5. Reference number 20 and 21 seems too old here presented as a basis for taking experiment.

*These references are used in the literature review to demonstrate the contrary conclusions drawn about the effect of SO_4_ ions. As we understand, this is still the state of the art. Additionally, we do not give special emphasis to that one anion in our work among the other anions that we tested in the matrix of salt types, i.e. this is not the basis for our experiment, but rather only support to the point that the picture is still unclear about anion effects.*

6. Line 82- working hypothesis is to enhance particle coagulation. I assume this can be extrapolated to aquatic systems. But, I am unable to find any solid reasons to justify as the soil and aquatic systems are totally different.

*We believe this comment of the reviewer is based on a bit of a mis-understanding. In this study we are not examining a soil system, but rather an artificially established water-based system that mimics a light water-based suspension of eroded soil particles. Such systems are the primary target of water quality research as a subject area. In practice, any successful and environmentally and economically feasible solution to further reduce particle and nutrient transport in such systems downstream could mean a minor break-through. Coagulation in such water-based system/solution is what we experimented with in laboratory conditions.*

7. Line 100- correct 13,9%.
8.Line 102- correct the spelling of course

*These are now rewritten.*

9.Line 105- explain the deviation observed in the protocol

*Thank you. We expanded on the nature of deviation by including the name of the chemical that was not applied to the solution.*

10. The statistical methods used for the experiment needs to be clear enough like see the presented data in table and figures are without SD and SE

*Following comments by both reviewers, we have enhanced the statistical analysis and expanded on its discussion. Please see our earlier response as well.*

11. Line 214 to 216 needs a recent reference.

*We now cite new literature here, also in line with the earlier comment on the SO_4_ anion.*

12. The discussion section just orients towards the science of coagulation with Monovalent and Divalent ions. Hence the authors must present novelty in this work.

*This manuscript reports about the first phase of research towards developing potentially new, applicable and acceptable technology to be used towards improving the quality of suface waters. In the literature, agriculture, and other land-based activities have been identified as major – if not the most dominant – polluters to surface waters world-wide. Reduction of the amount of soil sediment and nutrients dislodged from land and transported further downstream is a topic of extended research, with technologies and decision- and incentive systems of various kinds being sought to help increase retention efficiency. Our literature search showed that the potential use of coagulation-science coupled with environmentally present and acceptable coagulants is a majorly under-explored area in this direction, and we understand that this subject in general is still sufficiently novel.*

*We chose to approach this subject starting from a closed-environment laboratory experiment and discussion that:*

- *Quantifies the expectable effects of varied levels of available salt-type coagulants on sedimenting soil material, in attempt to report on the likely needed minimal amount of coagulant – by that helping to reduce future environmental impact when this research is taken to the field,*
- *Uses non-ideal material (i.e. real soil with its organic amendments) from a monitored, and historically erosion-prone local farm in a practically chosen concentration that aligns with literature-based (locally) historic-high amounts of transported sediment, and*
- *Discusses (improved after revisions) various aspects (practical, environmental, legal) of using the examined coagulants, and helps rule out certain salt choices for field application.*

*We believe that we compensate the currently limited scope of this work with sufficient evidence and practical choices that point towards good linkage to follow-up work in the field, yielding future impact. We attempted to include additional, modest references to aspects of novelty in the manuscript itself.*

13. They can discuss the ions individually. Please check the new references in that direction.

*We have added further references about the subject. However, for the same reasons that we have given earlier, we would refrain from expanding the discussion to concluding about individual ions. We believe that such discussion would increase the manuscript’s volume with rather repetitive text/conclusions, without real, practical benefit.*

14. Line 286- OH ions influences the pH. The authors should have presented the data for clarity. Speculations in the discussion section is partly acceptable.

*We have now added and discussed the OH- and pH relationship through providing the referred data, also in response to Reviewer #1.*

15. The reference section is not uniform. See Blankenberg et al and Sveistrup et al.
I hope to see these changes before final publication.

*We have corrected the reference list, thank you.*
