# Supplementary material for: The effect of various metal-salts on the sedimentation of soil in a water-based suspension
Source: PLoS One. 2020 Jan 14;15(1):e0227338. doi: 10.1371/journal.pone.0227338 (PMC6959554; doi:10.1371/journal.pone.0227338)
Supplement: Attachment — Submitted filename: Answer_to_editor.docx [file pone.0227338.s003.docx]

Dear Editor,

We appreciate your work and your comments to improve our article. We addressed all of your suggested modifications:

Comment 1&2: We changed all of the units to the form you marked.

Comment 3: In the description (L124-126) we add a sentence to clarify, why we did not test the marked salts.

Comment 4: The 'avg' expression changed to 'mean'.

Comment 5: We decreased the abstract and modified some sentence at L 10-17.

Comment 6: We divided the discussion into 3 smaller parts wit sub-headings.

We hope that these changes answer all of your questions.

Kind regards: The authors.
